# Antioxidant Properties of Bioactive Compounds in Fruit and Vegetable Waste

**DOI:** 10.3390/antiox12081647

**Published:** 2023-08-21

**Authors:** Nerea Jiménez-Moreno, Irene Esparza, Carmen Ancín-Azpilicueta

**Affiliations:** Department of Sciences, Institute for Advanced Materials (INAMAT^2^), Universidad Pública de Navarra, 31006 Pamplona, Spain

In recent years, great interest has arisen in the study of compounds with antioxidant activity present in agri-food residues. The growing demand from consumers for antioxidants of natural origin, the continuous growth in the world population and the consequent increase in the amount of food residues generated have greatly promoted this field of research. The search for new uses and applications for all this waste has become a mandatory objective within the framework of global environmental management. But, in addition, it offers a promising opportunity to study the great diversity of bioactive compounds that exist in nature and to analyze their biological activity and the associated health benefits. Among agri-food waste, fruit and vegetable by-products constitute a truly abundant and affordable source of natural antioxidants, such as phenolic compounds. These antioxidant phytochemicals are of great interest for different economic sectors, such as food, pharmaceutical and nutraceutical, the latter having seen an exponential development in recent years [1].

However, although antioxidant compounds are widespread in the plant kingdom, their concentrations are usually low. For this reason, it is very important to select those raw materials with the highest phytochemical content and those extraction methods that maximize recoveries, with low cost and low environmental impact [2]. In the last decade, numerous studies have analyzed the profile of bioactive compounds in different fruit and vegetable by-products, allowing for the selection of plant materials with a certain profile of compounds or with high contents of a specific phytochemical [3,4,5]. However, current research trends have to be driven beyond qualitative and quantitative analyses to characterize the raw plant matrices or the extracts obtained.

Focusing on the use of these antioxidant compounds in the food industry, it is necessary to know their behavior when they are added to a food that can undergo further processing or conservation treatments, since these treatments can alter their composition and quantity [6,7]. It is also important to analyze their stability under different conditions and to identify and study the biological action of the end products [8,9]. On the other hand, since the ultimate goal of using these plant by-products is to obtain bioactive compounds, it is very important to demonstrate their bioaccesibility during the digestion process. In general, the bioaccesibility of many phytochemicals is usually low, and sometimes their metabolites are responsible for exerting the antioxidant action on the organism [10,11]. On the other hand, during gastrointestinal digestion, the action of pH and of different enzymes can release other bioactive compounds that can be bound to other food components [12]. Finally, the role of the intestinal microbiota in the production of colonic metabolites, with important biological action, should not be underestimated [10]. Therefore, summarizing, for the compound or compounds of interest to exert a health benefit, they must resist food processing, be released from the food matrix after ingestion, be bioaccessible in the digestive tract, be metabolized and, finally, that compound or its biologically active metabolite must reach the target site [13]. Each of these requirements depends on different factors to be studied, so there is still a lot of work to be undertaken to shed light on the use of the antioxidant properties of the phytochemicals obtained from plant by-products.

This Special Issue includes six original research articles and two review articles that delve into different novel aspects of the extraction, bioaccesibility and use of bioactive phytochemicals present in fruit and vegetable waste. For instance, in the first original article, Ben Abdallah et al. [14] extracted hesperidin from orange by-products (80% peel and 20% pulp) using an ecofriendly and innovative extraction process. The optimized process combines instant controlled pressure drop (DIC) technology with an extraction technique called Tripolium. DIC technology is a pressure pretreatment, in which saturated steam (100–900 kPa) is applied to the raw material for less than 1 min, followed by a controlled pressure drop (>500 kPa/s) until reaching a final vacuum between 5 and 10 kPa. Combining this technique with the Tripolium extraction, which consists of the intermittent and successive application of an extraction solvent at moderate temperature and reduced pressure, increased the hesperidin recovery and the antioxidant capacity of the orange by-product extracts. DIC pretreatment generates a more expanded and porous material, improving the extraction efficiency. Moreover, this technology reduces the duration of both the raw material drying and the antioxidant extraction, as well as the energy requirements and the thermal degradation of phytochemicals.

In the work of Duarte et al. [15], the potential of almond bagasse, a poorly studied industrial by-product, was analyzed. The rise of vegetable drinks in recent years has produced significant amounts of new industrial by-products. These authors studied the influence of almond bagasse drying on the release of phenolic compounds in an in vitro simulation of the digestive process, as well as on the profile of the microbiota present in the colon. The results obtained indicated that drying the almond bagasse with hot air decreased the total content of polyphenols compared to the fresh by-product, unlike freeze-drying, which did not affect the phenolic content. However, both dehydration treatments significantly reduced the antiradical capacity of the extracts. Moreover, both dehydration treatments affected the release of phenolic compounds in different ways during the in vitro digestion of almond bagasse and had a clear effect on the composition of the in vitro fermentative microbiota, promoting the growth of beneficial bacteria for the host (*Butyrivibrio*, *Phascolarctobacterium*, *Dialister*). The results from these researchers suggest that the chemical and structural changes induced by dehydration treatments have an important influence beyond the extraction of bioactives from the raw material, since they also affect the interaction of digestive and microbial enzymes with this material. 

Díaz-Ramírez et al. [16] contributed to this Special Issue with a very comprehensive original article based on an integral valorization strategy for grape pomace using sustainable methodologies. These authors obtained cellulose nanocrystals (BCNCs) and polyphenol-rich extracts (GPPEs) both from grape pomace, and they combined them to develop a BCNC-GPPE complex. This complex has been shown to exert protective action on the extracted polyphenols, prolonging the half-life of their antioxidant properties. Additionally, these phenolic compounds modulated the surface charge of cellulosic nanocrystals, thus improving their colloidal properties. As a result, the BCNC-GPPE complex showed a great ability to stabilize Pickering emulsions, improving their oxidative stability. As the authors of this outstanding work conclude, the improved lipophilic affinity of the complex cellulose–polyphenolic extract opens up a wide range of promising applications, such as the development and application of food-grade Pickering emulsions, the recovery of polluting oils or the modulation of lipid digestion.

Grape pomace was also used in another article included in this Special Issue. In this case, Milinčić et al. [17] analyzed the bioaccessibility of phenolic compounds and the antioxidant properties of goat milk powder enriched with a grape pomace seed extract after in vitro gastrointestinal digestion. Their results seem to indicate that a large part of the phenolic compounds added with the extract to the milk would be found in the insoluble fraction that remains after gastrointestinal digestion, since the recovery of the extracted phenolics was very low. The authors suggested that it is necessary to examine this insoluble fraction in the future in order to use enriched powdered milk as a carrier of phenolics to the colon, where these compounds can be released and exert their antioxidant activity. In the work of Odriozola-Serrano et al. [18], however, the bioaccessibility of phenolic compounds from a hydroalcoholic extract of *Rosa canina* rosehips was studied at all stages of in vitro digestion, including the fermentative stage in the colon. The phenolic content and the antioxidant capacity of rosehip extracts significantly changed in the different digestion stages, probably due to the transformations that these phytochemicals can undergo and to the release of matrix-bound compounds during in vitro digestion. Flavonols and flavan-3-ols were highly bioaccessible and, therefore, they could easily exert their biological activity in the organism, while most phenolic acids were poorly bioaccessible and, thus, with much more limited biological effects. Finally, this original article pointed out the importance of analyzing the stability and bioaccessibility of phenolic compounds during the digestive process, in addition to their antioxidant capacity. Flavonoid glycosides showed lower antioxidant activity than aglycones, but their higher stability and bioaccessibility during in vitro digestion make them more interesting from a biological point of view.

Much of the research focused on the use of agri-food waste to obtain bioactive compound studies on the composition, biological activity and/or the applications of extractable compounds from fruit and vegetable waste, especially phenolic compounds. However, in agri-food by-products, phenolic compounds are generally in free and bound forms. The review article by Zeng et al. [19] highlighted the importance of non-extractable phenolic compounds, which are the bound phenolics that remain in the residual plant matrix after the extraction process. By neglecting the study of non-extractable phenolic compounds, the phenolic content and bioactivity of fruit and vegetable waste is underestimated since the non-extractable fraction accounts for a very important part of high-molecular-weight polymeric phenolic compounds or low-molecular-weight phenols chemically linked to macromolecules. This article points out the role of these compounds in mitigating cell damage caused by oxidative stress and reducing damage to the digestive tract mucosa. Due to the binding of non-extractable phenolics to macromolecules present in plant matrices, only a small fraction is released during gastrointestinal digestion, and it is upon reaching the colon when microbial fermentation transforms and/or releases them. For this reason, it is deeply important to investigate both secondary extraction processes, such as acid, alkaline or enzymatic hydrolysis, that favor the release of these compounds and pre-treatments of the raw material that make them more readily hydrolysable in the human body.

In the second review article, Sánchez et al. [20] compiled several valorization alternatives for cocoa bean shell, which is one of the main by-products derived from the chocolate industry. Cocoa bean shell is very rich in phenolic compounds, mainly flavanols, since during different processing steps in chocolate preparation, such as fermentation and roasting, these bioactive compounds migrate from the cocoa seed to the shell. However, in this work, the importance of bioactive peptide fractions present in cocoa bean shell with antioxidant activity is highlighted. In addition, very interesting and diverse alternatives to provide value to a waste that is generated annually in large quantities are collected. The encapsulation of extracts obtained from this by-product as a solution to protect and prolong the bioactivity half-life when added as an ingredient in different foods, the development of functional beverages or functional food packaging or even the development of new foods, such as cocoa tea, are some of them. Likewise, although marginally related to the topic of the Special Issue, other very interesting options are analyzed, such as the use of cocoa bean shell as an adsorbent to eliminate drugs or several types of dye from water, as new energy alternatives (bioethanol, pellets) or even as a corrosion inhibitors for carbon steel.

Finally, Chumroenvidhayakul et al. [21] added dragon fruit peel powder to wheat cookies in order to reduce the glycemic index and the formation of toxins from lipid peroxidation and dietary advanced glycation end products (known as dAGE) in this food product. Dragon fruit peel is a residue generated largely in Thailand and is very rich in bioactive compounds, such as betacyanins, phenolic compounds and dietary fiber. The addition of dragon fruit peel powder to the cookies reduced the level of toxins produced during the thermal treatment, probably because the dietary fiber and bioactive phytochemicals present in this by-product interfere in the reactions responsible for the formation of dAGEs (caramelization, Maillard’s reaction and lipid peroxidation). Likewise, these components of the dragon fruit peel can also interfere with the formation of the starch gel, thus reducing the digestibility of this carbohydrate. In addition, sensory analysis, including appearance, color, odor, taste, texture, hardness and the overall acceptance of cookies with dragon fruit peel powder, showed that an addition of up to 2% (*w*/*w*) exhibits a high sensory acceptance. These results offer a promising opportunity for the food industry: to prepare healthier bakery foods using powder obtained from fruit and vegetable by-products, without the need for a prior extraction process.

There is still a long way to go in research on the potential uses of fruit and vegetable wastes with antioxidant properties. Future research in this field should be driven towards the search for specific applications for each type of plant by-product based on both its physicochemical characteristics and major components. Likewise, since the objective is to obtain a health benefit from the phytochemicals present in fruits and vegetables, it is of vital importance to acquire a complete and in-depth knowledge about the fate of these bioactive compounds, either during food processing or during digestion and gastrointestinal fermentation, as well as all the factors that can influence these aspects of bioactive compounds.

Finally, we would like to express our gratitude to all the authors who participated in this Special Issue and to the reviewers for their great work. Many thanks also to the *Antioxidants* journal for giving us the opportunity to be Guest Editors of this Special Issue.
